# Factors associated with infant and young child feeding practices in Kaduna and Lagos States, Nigeria

**DOI:** 10.1371/journal.pgph.0004753

**Published:** 2025-06-27

**Authors:** Valerie L. Flax, Mariam Fagbemi, Carrie Ngongo, Susan Edwards, Jigna M. Dharod, Victor Ogbodo

**Affiliations:** 1 RTI International, Durham, North Carolina, United States of America; 2 Yucca Consulting, Lagos, Nigeria; 3 University of North Carolina Greensboro, Greensboro, North Carolina, United States of America; 4 Alive & Thrive, FHI 360, Abuja, Nigeria; Michigan State University, UNITED STATES OF AMERICA

## Abstract

Early initiation of breastfeeding (EIBF), exclusive breastfeeding (EBF), and minimum dietary diversity (MDD) are recommended infant and young child feeding (IYCF) practices. Subnational analyses of predictors of these practices are rare but can contribute to programmatic and policy decisions. The aim of this study was to assess predictors of EIBF, EBF, and MDD in Kaduna and Lagos States, Nigeria. This analysis of the population-based data from the Alive & Thrive impact evaluation [Kaduna (N = 4,702), Lagos (N = 1,563)] used chi-square automatic interaction detection to select variables related to EIBF, EBF, and MDD with P < 0.20. Final logistic regression models retained predictors associated with each outcome at P < 0.05. Positive predictors of EIBF were woman-headed household (both states), contact with a health worker (both), EIBF knowledge (both), EIBF awareness (Lagos), EIBF beliefs (both), EIBF self-efficacy (Kaduna), maternal education (Kaduna), household food security (Kaduna), delivery at health facility (Kaduna), counseling on EIBF (Lagos), and vaginal birth (Lagos). Maternal employment was negatively associated with EIBF (Kaduna). Positive predictors of EBF were EBF knowledge (both), EBF awareness (Kaduna), EBF beliefs (Kaduna), EBF self-efficacy (both), postpartum practices such as checking to see if the baby was sucking well (Kaduna), maternal education (Kaduna), household food security (Kaduna), and EBF norms (Lagos). The child having fever in the last 2 weeks was negatively associated with EBF (Kaduna). Positive predictors of MDD were child’s age (both), socioeconomic status (both), four or more antenatal care visits (Kaduna), child still breastfed (Kaduna), complementary feeding awareness (Kaduna), complementary feeding beliefs (Kaduna), help from husband or mother/mother-in-law (Kaduna), contact with a health worker (Lagos), complementary feeding knowledge (Lagos), and complementary feeding self-efficacy (Lagos). Maternal education (Kaduna) and household food security (Lagos) were negatively associated with MDD. This study reveals that different combinations of behavioral factors and maternal and household characteristics are critical predictors of IYCF in Nigeria, though variations exist between Kaduna and Lagos. These insights can inform tailored state-specific IYCF interventions and policies to improve IYCF practices in Nigeria.

## Introduction

Optimal infant and young child feeding (IYCF) practices facilitate child survival, growth, and development [1]. Given these benefits, the World Health Organization (WHO) recommends that children start breastfeeding within 1 hour of delivery (early initiation), breastfeed exclusively until 6 months of age, and thereafter consume a minimally diverse diet that includes at least five of eight food groups daily [2]. Many countries face challenges increasing the percentage of infants and young children who are fed according to these guidelines. In sub-Saharan Africa (SSA) during the period from 2016-2022, IYCF indicator prevalence was 53% for early initiation of breastfeeding (EIBF), 48% for exclusive breastfeeding (EBF), and 23% for minimum dietary diversity (MDD) [3]. In Nigeria, data from 2023-24 indicated that EIBF (36%), EBF (29%), and MDD (12%) prevalence estimates were lower than SSA averages [4]. Within Nigeria, IYCF practices can vary substantially by state, but there is limited research examining these differences or the factors that influence the variability in IYCF sub-nationally. Understanding the factors that predict IYCF practices sub-nationally can help government entities and their partners to design tailored child nutrition programs and interventions.

A variety of factors influence IYCF practices in SSA, including household socioeconomic status, maternal characteristics, child characteristics, use of health services, and receipt of information and support. For example, factors positively associated with EIBF range from maternal education to birth circumstances, including delivery in a health facility, vaginal birth, larger birth size, and health status of the baby [5–14]. Evidence is mixed regarding the association of maternal marital status [9,10] and birth order [9,11,15] with EIBF. Exposure to breastfeeding counseling and antenatal care are positively associated with EIBF [6,7,10,16,17].

EBF is associated with marital status, maternal education, age, parity, wealth, social support, and breastfeeding counseling [18–23]. As with EIBF, infants with normal or high birth weight, delivered vaginally, and delivered in health facilities are more likely to be exclusively breastfed [20,22–24]. Mothers are less likely to exclusively breastfeed if they work outside the home, perceive that they have insufficient milk supply, or receive breastfeeding guidance from sources other than health professionals [21,25,26].

Child and maternal age are associated with MDD, alongside maternal education, employment or self-employment, mass media exposure, wealth, and availability of money to buy food [27–38]. Children who access health facilities and are vaccinated are more likely to achieve MDD [34,35,38]. Households with collaborative decision-making on household spending, with children spaced at least 24 months apart, and where the child is fed using a separate plate are more likely to provide MDD [36]. MDD is higher in urban settings than in rural and peri-urban areas [30,36]. Less work has been done to understand the determinants of MDD as compared with breastfeeding practices.

Previous studies assessing factors associated with IYCF practices have several limitations. Cross-sectional studies often are geographically constrained with small sample sizes [6,7,10,17,18,20–23,25,26,28,30,36,37,39,40]. Some studies focus on only a single predictor [12,29,40]. Studies using national survey data can provide estimates for one or more countries, but they typically are limited in the types of variables that can be included and do not assess subnational variability [5,8,9,11,13–16,19,24,27,29,31–35,38]. Limited evidence is available regarding the association of counseling and maternal knowledge, attitudes, self-efficacy, and intentions with IYCF practices.

Very few studies of IYCF predictors have been conducted in Nigeria. We found two small studies of predictors of EBF in Lagos (N = 120) [20] and Ido-Ekiti (N = 217) [18] and one study that used Nigeria Demographic and Health Survey data to assess the effect of cesarean section on EIBF [12]. This indicates that in addition to the limitations in studies of IYCF in SSA, there are broader evidence gaps in Nigeria, including small sample sizes, few predictors of EIBF, no studies of predictors of MDD, and no comparisons by state.

The main aim of this study was to identify predictors of three IYCF practices—EIBF, EBF, and MDD—in two states of Nigeria. The secondary aim was to understand the differences in predictors between two settings with different sociocultural contexts. The data analyzed were collected as part of a program impact evaluation with state-level sampling and a diversity of variables not available in large national datasets.

## Materials and methods

### Study setting

This analysis used secondary data from the impact evaluation baseline of the Alive & Thrive IYCF program in Kaduna and Lagos States, Nigeria [41]. These two states were selected for the first phase of Alive & Thrive implementation in Nigeria because their contrasting demographic, socioeconomic, and cultural settings allowed for broad learning on which to base later program scaling. This state-level variability was used in the present study to explore whether predictors of IYCF practices differ sub-nationally.

Kaduna State is in northwestern Nigeria and has a population of approximately 9 million, with about 20% living in urban areas [42]. Kaduna has a mix of Muslims and Christians and more than 60 ethnic groups, the most prominent being Hausa and Gbagyi. In Kaduna, about 75% of women receive antenatal care, but more than 80% of women deliver their babies at home [4]. Lagos State is a large urban area on the southwest coast of Nigeria with a population of more than 15 million; although by some estimates it has up to 25 million inhabitants [43]. Lagos is the economic capital of Nigeria, and its inhabitants include all the major ethnic groups, with Yoruba and Pidgin as the main languages. In Lagos, almost all women receive antenatal care and more than 80% deliver in a health facility, with nearly half delivering in a private health facility [4]. It is common to introduce water to infants early in both states, whereas prelacteal feeding is more common in Kaduna [44].

### Participants

Women were eligible for the study if they had a biological child who was 0–23 months old. They could participate if they were over 18 years old, regardless of marital status, or 15–17 years old and married. Under the Nigerian constitution, 18 years is the age of majority and younger married women are considered to be of full age [45].

### Data collection

All 23 local government areas (LGAs) in Kaduna and 16 of 20 LGAs in Lagos were included in the study. Population-based surveys were conducted in both states. Geo-sampling was used as the primary method by putting a grid over the map of each LGA. Primary sampling units were 1 km^2^, and the size of secondary sampling units was based on whether the area was urban (50 m^2^), semiurban (100 m^2^), or rural (150 m^2^). Primary and then secondary sampling units were randomly sampled, and all households within the selected secondary sampling units were screened and invited to participate if eligible. In 9 LGAs where we could not obtain an adequate sample in some subgroups using geo-sampling, random route walks were performed in administrative wards that were not covered by the geo-sample to boost subgroup samples. Our random route walk procedure used a modified multistage random sampling technique where a random sample of wards not used for geo-sampling were selected followed by a further random sample of two sectors within each of those wards. Starting points for the walks were randomized based on the day’s date, and dwelling units were selected using a skip pattern based on the population density of the area.

The questionnaire was developed in English and translated into Hausa and Yoruba by professional translators in Nigeria. The translations were further refined following review by the Alive & Thrive Nigeria team, cross checking across languages for accuracy, and pilot testing of the questionnaire during interviewer training. The questionnaire included the WHO IYCF questionnaire [2] and questions on household and individual socioeconomic characteristics and other factors that might influence IYCF practices, such as use of health services; location of delivery; IYCF knowledge, attitudes, beliefs, norms, and self-efficacy; child morbidity; and support or assistance from health workers and family members. Questions on socioeconomic characteristics and child morbidity were taken from the Nigeria Demographic and Health Survey questionnaire [4]. Questions on IYCF knowledge, attitudes, beliefs, norms, and self-efficacy were adapted from Alive & Thrive evaluations in other countries.

Experienced interviewers from the two states who spoke either Hausa or Yoruba participated in a 5-day training that covered the aims of the study, sampling procedures, data collection procedures, and research ethics. They recruited mothers and administered the questionnaire to them in their homes using the NField tablet-based data collection system. The survey took place from January to July 2017.

### Ethics

Mothers gave verbal informed consent for themselves and their child. Interviewers read the consent form aloud to mothers and documented their verbal consent in the NField data collection system. The verbal consent procedure was approved by all ethics committees. Ethical approval was granted by institutional review boards at RTI International and FHI 360 and the ethics committees of the Kaduna State Ministry of Health and the Lagos State Government through the Lagos State University Teaching Hospital, Ikeja. A protocol amendment was obtained for the random route walk sampling approach that was used to complete the sample in some LGAs. The present secondary analysis used deidentified data available on Harvard Dataverse: https://doi.org/10.7910/DVN/ZI1HUO.

### Variable definitions

The three outcome variables were IYCF practices, including EIBF, EBF, and MDD, and created following the WHO IYCF indicator guidelines. EIBF was defined as starting breastfeeding within 1 hour of birth, assessed for children 0–23 months. EBF was defined as consumption of only breastmilk on the previous day, assessed for children 0–5 months. MDD was defined as consumption of at least five of eight food groups on the previous day, assessed for children 6–23 months. All predictor variables considered for modeling are defined in Table 1. Predictors included maternal factors and characteristics, antenatal care and delivery characteristics, household and socioeconomic factors, child factors, knowledge and behavioral factors, and mother’s complementary food decision making. S1 Table provides survey questions used in the composite scores for each of the knowledge and behavioral factors.

**Table 1:** Predictor variables, definitions, and predictors tested by IYCF practice.

Variable	Definition	Tested as possible predictors		
		EIBF	EBF	MDD
**Maternal factors and characteristics**				
Works at home or away from home	0 = Works away from home part-time or full-time 1 = Works at home	X	X	X
Marital status	0 = Not married (single, separated, divorced, widowed) 1 = Married	X	X	X
Employment status	0 = Does not work in paid employment 1 = Works in paid employment	K	X	X
Education level	1 = Never attended school 2 = Some primary 3 = Completed primary 4 = Some secondary 5 = Completed secondary or higher	K	K	K
**Antenatal care and delivery characteristics**				
Number of antenatal care visits during pregnancy with child	0 = None 1 = 1–3 visits 2 = 4 or more visits	K	X	K
Delivery location	0 = At home 1 = In a health care facility	K	X	X
Vaginal birth	0 = No 1 = Yes	L	–	–
Received formal assistance with delivery	0 = No 1 = Yes	X	–	–
**Household and socioeconomic factors**				
Mother is head of household	0 = No 1 = Yes	KL	X	X
Household food insecurity	0 = Mildly to severely food insecure 1 = Food secure	K	K	L
Socioeconomic status	Principal components analysis of main source of drinking water, type of toilet facility, main floor material, main roof material, main exterior wall material, household item asset count	X	K	KL
**Exposure to IYCF counseling/messaging**				
Received any counseling on IYCF outcome topic	0 = No 1 = Yes	L	X	X
Had any contact with a community health extension worker or community volunteer where IYCF was discussed in last 6 months	0 = No 1 = Yes	KL	X	L
**Social support for IYCF**				
From husband/partner	0 = No 1 = Yes	X	X	K
From mother/mother-in-law	0 = No 1 = Yes	X	K	K
**Child factors**				
Child’s age in months	Continuous	–	–	KL
Child appetite	0 = Appetite is not good 1 = Appetite is good or very good	–	–	X
Fever in the past 2 weeks	0 = No 1 = Yes	–	K	X
Cough/cold in the past 2 weeks	0 = No 1 = Yes	–	–	X
Diarrhea in the past 2 weeks	0 = No 1 = Yes	–	X	X
Still breastfeeding	0 = No 1 = Yes	–	–	K
**Knowledge and behavioral factors** ^1^				
Knowledge composite score	EIBF – 2 questions EBF – 4 questions MDD – 9 questions	KL	KL	L
Awareness composite score	EIBF – 2 questions EBF – 1 question MDD – 2 questions	L	K	K
Beliefs composite score	EIBF – 2 questions EBF – 8 questions MDD – 2 questions	KL	K	K
Norms composite score	EIBF – 1 question EBF – 7 questions MDD – 2 questions	X	L	X
Self-efficacy composite score	EIBF – 2 questions EBF – 3 questions MDD – 2 questions	K	KL	L
Postpartum practices composite score (such as avoiding prelacteal feeding and checking how well the baby is sucking)	EIBF – 2 questions EBF – 3 questions	KL	K	–
**Mother’s complementary food decision making composite score**	MDD – 2 questions	–	–	X

^1^Questions used to create composite scores are shown in S1 Table.

X = variable tested as a predictor but not included in final model: K = variable included in final Kaduna model; L = variable included in final Lagos model; Dash = variable not tested for that IYCF outcome.

### Analysis

The dataset contained information on multiple factors related to each IYCF practice. Categorical factors were collapsed when possible if a level of the factor did not have sufficient numbers for modeling (e.g., single, separated, divorced, and widowed were collapsed into “not married”). Principal components analysis was used to create continuous score variables for socioeconomic status and timing of introduction of new foods. Composite scores were created for mother’s knowledge, awareness, beliefs, norms, self-efficacy, and postpartum practices by summing responses to questions specific to each of the outcomes. In the analysis, an item in the composite scores refer to a positive response to a question within the score. The number of items in the composite scores varied across behavioral factors and IYCF practices.

To further reduce related variables in each model, chi-square automatic interaction detection (CHAID), a non-parametric method, was used to identify key factors within a factor group (e.g., all variables related to maternal or household factors) to consider for the final models. Specifically, CHAID identified categorical variables related to each IYCF practice with a p-value < 0.20 and identified similar levels within a categorical variable with a p-value > 0.20. When possible, levels within a categorical variable were further collapsed without losing meaningful information based on the CHAID results. The identified variables were considered to be the best representation of the overall factor and used in the base model for each IYCF practice.

After using CHAID to identify the key variables that represented the relationship between an overall factor and the IYCF practice, an additional CHAID, with the same statistical significance thresholds, was used to identify interaction terms for the base model. The final logistic regression models with interaction terms for each IYCF practice were determined using backward selection, retaining predictors associated with each outcome at the p < 0.05 level of statistical significance. All analysis was conducted with Stata 17.0.

## Results

### Sample characteristics

Household and participant characteristics of the sample have previously been reported [41]. Briefly, in Kaduna, the mean child’s age was 9 months, 51% of the children were male, 40% of mothers had never attended school, the mean mother’s age was 27 years, 98% of mothers were married, and about 55% were small traders or self-employed. In Lagos, the mean child’s age was approximately 8.5 months, 49% of the children were male, the mean mother’s age was 30 years, more than 85% of mothers had completed secondary school or higher, 97% of mothers were married, and about 68% were small traders or self-employed.

### Distribution of outcome and predictor variables

The percentage distribution of all predictors retained in the final models is included for each outcome by state in Table 2. The unweighted prevalence of the IYCF outcome variables was 34.4% and 41.5% for EIBF; 27.4% and 56.8% for EBF; and 16.3% and 26.8% for MDD in Kaduna and Lagos, respectively. Socioeconomic, maternal, and child characteristics differed by state. A higher percentage of mothers in Lagos were employed, had secondary education or higher, attended four or more antenatal visits, delivered in a health facility, received counseling on IYCF, and received help from their mother/mother-in-law. A higher percentage of children in Kaduna were still breastfed from 6 to 23 months and had fever in the last 2 weeks. Mothers in Lagos generally had higher knowledge, awareness, beliefs, norms, and self-efficacy scores related to the three IYCF practices than those in Kaduna.

**Table 2:** Unweighted percentage distribution of outcome and predictor variables.

Characteristic	EIBF		EBF		MDD	
	Kaduna	Lagos	Kaduna	Lagos	Kaduna	Lagos
	N (%)	N (%)	N (%)	N (%)	N (%)	N (%)
	N = 4702	N = 1563	N = 1787	N = 646	N = 2916	N = 917
**Early initiation of breastfeeding**	1616 (34.4)	649 (41.5)				
**Exclusive breastfeeding**			490 (27.4)	367 (56.8)		
**Minimum dietary diversity**					474 (16.3)	246 (26.8)
**Mother’s employment status**						
Not employed (ref)	1607 (34.2)	297 (19.0)	701 (39.2)	118 (18.3)	906 (31.1)	179 (19.5)
Employed	3095 (65.8)	1266 (81.0)	1086 (60.8)	528 (81.7)	2010 (68.9)	738 (80.5)
**Mother’s education**						
No education (ref)	1885 (40.1)	32 (2.1)	701 (39.3)	6 (0.9)	1184 (40.7)	26 (2.8)
Some primary	531 (11.3)	31 (2)	186 (10.4)	11 (1.7)	345 (11.8)	20 (2.2)
Completed primary	487 (10.4)	118 (7.6)	178 (10)	36 (5.6)	309 (10.6)	82 (9)
Some secondary	655 (13.9)	133 (8.5)	242 (13.6)	55 (8.5)	413 (14.2)	78 (8.5)
Secondary or higher	1138 (24.2)	1246 (79.9)	478 (26.8)	537 (83.3)	661 (22.7)	709 (77.5)
**Mother is household head**	264 (5.6)	165 (10.6)	120 (6.7)	102 (15.8)	145 (5.0)	63 (6.9)
**Household food security**						
Mildly to severely food insecure (ref)	2157 (46.8)	676 (44.6)	732 (41.7)	257 (40.5)	1425 (49.9)	419 (47.5)
Food secure	2453 (53.2)	841 (55.4)	1022 (58.3)	377 (59.5)	1431 (50.1)	464 (52.5)
**Antenatal care**						
0–3 visits (ref)	1497 (33.3)	55 (4.1)	587 (34.1)	14 (2.5)	909 (32.7)	41 (5.1)
4 + visits	2999 (66.7)	1300 (95.9)	1132 (65.9)	542 (97.5)	1868 (67.3)	758 (94.9)
**Location of delivery**						
At home (ref)	3325 (70.7)	120 (7.7)	1231 (68.9)	45 (7.0)	2094 (71.8)	75 (8.2)
In a health facility	1377 (29.3)	1443 (92.3)	556 (31.1)	601 (93.0)	822 (28.2)	842 (91.8)
**Vaginal birth (not cesarean)**	4584 (97.5)	1400 (89.6)				
**Received counseling on outcome (EIBF/EBF/MDD)**	438 (9.3)	293 (18.7)	1297 (72.6)	593 (91.8)	1649 (56.6)	766 (83.5)
**Had contact with health worker in last 6 months**	213 (4.6)	69 (4.4)	66 (3.7)	23 (3.6)	147 (5.1)	46 (5.1)
**Any help from mother/mother-in-law**	2532 (53.8)	1216 (77.8)	911 (51.0)	527 (81.6)	1622 (55.6)	689 (75.1)
**Any help from husband**	4238 (90.1)	1499 (95.9)	1604 (89.8)	629 (97.4)	2635 (90.4)	870 (94.9)
**Child is still breastfed**					2310 (79.3)	574 (62.7)
**Child had fever in last 2 weeks**			461 (25.8)	30 (4.6)	1411 (48.4)	164 (17.9)
**EIBF knowledge score**						
0 items (ref)	421 (9)	104 (6.7)				
1 item	2277 (48.4)	615 (39.3)				
2 items	2004 (42.6)	844 (54.0)				
**EIBF beliefs score**						
0 items (ref)	733 (15.6)	315 (20.2)				
1 item	256 (5.4)	120 (7.7)				
2 items	3713 (79.0)	1128 (72.2)				
**EIBF self-efficacy score**						
0 items (ref)	468 (10.0)	81 (5.2)				
1 item	985 (20.9)	244 (15.6)				
2 items	3249 (69.1)	1238 (79.2)				
**EBF knowledge score**						
0–2 items (ref)			1302 (72.9)	264 (40.9)		
3–4 items			485 (27.1)	382 (59.1)		
**EBF awareness**			1054 (59.0)	613 (94.9)		
**EBF beliefs score**						
0–2 items (ref)			377 (21.1)	79 (12.2)		
3–4 items			880 (49.2)	265 (41.0)		
5–8 items			530 (29.7)	302 (46.7)		
**EBF norms score**						
0–4 items (ref)			1534 (85.8)	377 (58.4)		
5 items			83 (4.6)	69 (10.7)		
6–7 items			170 (9.5)	200 (31.0)		
**EBF self-efficacy score**						
0–1 items (ref)			626 (35.0)	124 (19.2)		
2 items			436 (24.4)	139 (21.5)		
3 items			725 (40.6)	383 (59.3)		
**Postpartum practices score**						
0 items	560 (11.9)	68 (4.4)	183 (10.2)	18 (2.8)		
1 item	2035 (43.3)	583 (37.3)	783 (43.8)	254 (39.3)		
2 items	2108 (44.8)	912 (58.3)	653 (36.5)	308 (47.7)		
3 items			168 (9.4)	66 (10.2)		
**Complementary feeding knowledge score**						
0–4 items (ref)					2229 (76.4)	442 (48.2)
5–9 items					687 (23.6)	475 (51.8)
**Complementary feeding awareness score**						
0 items (ref)					1568 (53.8)	47 (5.1)
1 item					607 (20.8)	148 (16.1)
2 items					741 (25.4)	722 (78.7)
**Complementary feeding beliefs score**						
0–1 item (ref)					551 (18.9)	222 (24.2)
2 items					2365 (81.1)	695 (75.8)
**Complementary feeding self-efficacy score**						
0 items (ref)					1065 (36.5)	185 (20.2)
1–2 items					1851 (63.5)	732 (79.8)

### Predictors of EIBF

Maternal educational attainment was positively associated with EIBF in Kaduna, including some primary school (odds ratio [OR] 1.31), completed primary to some secondary (OR 1.41), and secondary or higher (OR 1.86) (Table 3). Household factors were positively associated with EIBF, including the mother being the household head (Kaduna OR 1.51, Lagos OR 3.11) and the household being food secure (Kaduna OR 1.40). Health facility birth (Kaduna OR 1.41), vaginal birth (Lagos OR 7.17), maternal contact with a health worker in the last 6 months (Kaduna OR 1.47, Lagos OR 2.07), and counseling on EIBF (Lagos OR 1.26) were positively associated with EIBF. Behavioral factors were also positively associated with EIBF, including knowledge of EIBF (Kaduna 2 items OR 12.69; Lagos 1 item OR 2.79, 2 items OR 13.62), EIBF awareness (Lagos 1 item OR 1.32), EIBF beliefs (Kaduna 1 item OR 1.90, 2 items OR 1.71; Lagos 1-2 items OR 1.84), and EIBF self-efficacy (Kaduna 2 items OR 2.70). Maternal employment (Kaduna OR 0.82) was the only factor that was negatively associated with EIBF. In Kaduna, there were significant interactions between mother’s knowledge score and three other variables (number of antenatal care visits, EIBF self-efficacy, and postpartum practices score) and between EIBF self-efficacy and food security (S2 Table). In Kaduna, mothers with more EIBF knowledge (2 of 2 items) and three or fewer antenatal visits were more likely to practice EIBF than those with four or more antenatal visits, although knowledge was strongly associated in both cases (≤3 visits OR 12.69, 4 + visits OR 8.98). Mothers in Kaduna with one item of EIBF knowledge were more likely to practice EIBF if they also practiced one postpartum practice. ORs were constant across postpartum practices for mothers with zero or two item(s) of EIBF knowledge. Mothers with no EIBF knowledge were more likely to practice EIBF if they scored two items for EIBF self-efficacy. Mothers with two items for EIBF self-efficacy were more likely to practice EIBF if they lived in a food-secure household.

**Table 3:** Factors predicting early initiation of breastfeeding among children 0-23 months in Kaduna and Lagos.

Predictors	Kaduna	Lagos
	OR (95% CI)	OR (95% CI)
**Mother’s education**		
No education (ref)	.	
Some primary	1.31 (1.02, 1.68)	
Completed primary to some secondary	1.41 (1.16, 1.71)	
Secondary or higher	1.86 (1.51, 2.30)	
**Mother’s employment status**		
No employment (ref)	.	
Employment	0.84 (0.73, 0.98)	
**Mother is household head**	1.51 (1.13, 2.02)	3.11 (2.08, 4.64)
**Household food security**		
Mildly to severely food insecure (ref)	.	
Food secure	1.55 (0.93, 2.57)	
**Delivery location**		
At home (ref)	.	
In a health facility	1.41 (1.19, 1.67)	
**Antenatal care**		
0–3 visits (ref)	.	
4 + visits	1.22 (0.67, 2.23)	
**Vaginal birth (not cesarean)**		7.17 (4.37, 11.76)
**Contact with health worker in last 6 months**	1.47 (1.07, 2.04)	2.07 (1.18, 3.66)
**Counseling on EIBF**		2.16 (1.59, 2.93)
**EIBF knowledge score**		
0 items (ref)		.
1 item	0.95 (0.44, 2.06)	2.79 (1.30, 6.03)
2 items	12.69 (6.86, 23.50)	13.62 (6.33, 29.30)
**EIBF awareness score**		
0 items (ref)		.
1 item		1.32 (1.02, 1.70)
**EIBF beliefs score**		
0 items (ref)	.	.
1 item (Kaduna)	1.90 (1.31, 2.75)	
2 items (Kaduna)	1.71 (1.35, 2.18)	
1–2 items (Lagos)		1.84 (1.29, 2.61)
**EIBF self-efficacy score**		
0 items (ref)		
1 item	0.86 (0.55, 1.35)	
2 items	2.70 (1.38, 5.29)	
**Postpartum practices score**		
0 items	.	
1 item	0.97 (0.68, 1.38)	
2 items	0.99 (0.70, 1.40)	

In Kaduna, there were significant two-way interactions between mother’s EIBF knowledge score and number of antenatal care visits, mother’s EIBF self-efficacy score, and postpartum practices score and between mother’s EIBF self-efficacy score and food security. See S2 Table for ORs for interactions.

### Predictors of EBF

Some secondary or higher maternal education (OR 1.51) and household food security (OR 1.35) were associated with EBF in Kaduna (Table 4). Among behavioral factors, knowledge of EBF (Kaduna OR 4.40, Lagos OR 1.71), EBF awareness (Kaduna OR 2.02), EBF beliefs (Kaduna 3–4 items OR 1.56, 5–8 items OR 1.72), EBF norms (Lagos 5 items OR 1.81, 6–7 items OR 8.25), EBF self-efficacy (Kaduna 2 items OR 1.84, 3 items OR 3.19; Lagos 2 items OR 2.02, 3 items OR 4.23), and postpartum practices (Kaduna 2–3 items OR 1.82) were positively associated with EBF. The only factor negatively associated with EBF was the child having a fever in the last 2 weeks (Kaduna OR 0.69). In Kaduna, there were significant interactions between help from mother/mother-in-law and mother’s EBF knowledge and between EBF self-efficacy score and EBF beliefs score (S3 Table). Mothers who did not receive help from their mother/mother-in-law and who had three or more correct EBF knowledge items were more likely to practice EBF. Mothers with more EBF belief items and more self-efficacy items were more likely to practice EBF.

**Table 4:** Factors predicting exclusive breastfeeding among children 0-5 months in Kaduna and Lagos.

Predictors	Kaduna	Lagos
	OR (95% CI)	OR (95% CI)
**Mother’s education**		
None or primary (ref)	.	
Some secondary or higher	1.51 (1.13, 2.02)	
**Household food security**		
Mildly to severely food insecure (ref)	.	
Food secure	1.35 (1.03, 1.78)	
**Socioeconomic status**	1.21 (1.08, 1.36)	
**Child had fever in last 2 weeks**	0.69 (0.50, 0.94)	
**EBF knowledge score**		
0–2 items (ref)	.	.
3–4 items	4.40 (2.87, 6.77)	1.71 (1.18, 2.48)
**EBF awareness**	2.02 (1.45, 2.81)	
**EBF beliefs score**		
0–2 items (ref)	.	
3–4 items	1.56 (1.02, 2.39)	
5–8 items	1.72 (1.01, 2.92)	
**EBF norms score**		
0–4 items (ref)		.
5 items		1.81 (1.05, 3.13)
6–7 items		8.25 (5.02, 13.58)
**EBF self-efficacy score**		
0–1 items (ref)	.	.
2 items	1.84 (1.24, 2.73)	2.02 (1.15, 3.55)
3 items	3.19 (2.14, 4.77)	4.23 (2.59, 6.90)
**Postpartum practices score**		
0–1 items (ref)	.	
2–3 items	1.82 (1.40, 2.37)	
**Any help from mother/mother-in-law**	1.15 (0.82, 1.60)	

In Kaduna, there were significant two-way interactions between help from mother/mother-in-law and mother’s EBF knowledge score and between mother’s EBF self-efficacy score and mother’s EBF beliefs score. See S3 Table for ORs for interactions.

### Predictors of MDD

Use of health services was positively associated with MDD, including four or more antenatal care visits (Kaduna OR 1.51) and any contact with a health worker in the last 6 months (Lagos OR 2.46) (Table 5). Socioeconomic status (Kaduna OR 1.21, Lagos OR 1.38), child’s age (Kaduna OR 1.14, Lagos OR 1.02), and child is still breastfed (Kaduna OR 1.72) were also positively associated with MDD. Several behavioral factors were positively associated with MDD, including complementary feeding knowledge (Lagos 5–9 items OR 1.47), complementary feeding awareness (Kaduna 1 item OR 1.94, 2 items OR 2.01), complementary feeding beliefs score (Kaduna 2 items OR 1.36), and complementary feeding self-efficacy (Lagos 1–2 items OR 3.79). In Kaduna, any help from husband (OR 1.76) or from mother/mother-in-law (OR 1.31) was also positively associated with MDD. Factors that were negatively associated with MDD were some secondary or higher maternal education (Kaduna OR 0.74) and household food security (Lagos OR 0.67). In Kaduna, there was a significant interaction between mother’s education and still breastfeeding (S4 Table). Women who were still breastfeeding were more likely to have a child with MDD, regardless of education level. Women who were not breastfeeding and had higher education were less likely to have a child with MDD.

**Table 5:** Factors predicting minimum dietary diversity among children 6-23 months in Kaduna and Lagos.

Predictors	Kaduna	Lagos
	OR (95% CI)	OR (95% CI)
**Mother’s education**		
None or primary (ref)	.	
Some secondary or higher	0.74 (0.46, 1.17)	
**Household food security**		
Mildly to severely food insecure (ref)		.
Food secure		0.67 (0.49, 0.92)
**Antenatal care**		
0–3 visits (ref)	.	
4 + visits	1.51 (1.15, 1.98)	
**Any contact with health worker in the last 6 months**		2.46 (1.26, 4.79)
**Socioeconomic status**	1.21 (1.11, 1.33)	1.38 (1.20. 1.57)
**Child’s age (months)**	1.14 (1.11, 1.17)	1.02 (0.99, 1.05)
**Child is still breastfed**	1.72 (1.15, 2.58)	
**Complementary feeding knowledge score**		
0–4 items (ref)		.
5–9 items		1.47 (1.08, 2.02)
**Complementary feeding awareness score**		
0 items (ref)	.	
1 item	1.94 (1.46, 2.56)	
2 items	2.01 (1.54, 2.62)	
**Mother’s complementary feeding beliefs score**		
0 or 1 item (ref)	.	
2 items	1.36 (1.00, 1.87)	
**Mother’s complementary feeding self-efficacy**		
0 items (ref)		.
1–2 items		3.79 (1.77, 8.15)
**Any help from husband**	1.76 (1.07. 2.89)	
**Any help from mother/mother-in-law**	1.31 (1.05, 1.65)	

In Kaduna, there was a significant two-way interaction between mother’s education and still breastfeeding. See S4 Table for ORs for interactions.

## Discussion

This analysis identified various factors that were associated with EIBF, EBF, and MDD in Kaduna and Lagos States. Several factors were associated with EIBF in both states, including the mother’s status as household head, contact with a health worker, knowledge, and beliefs. The factors that differed by state likely reflect contextual differences. For example, in Kaduna, fewer women have formal education, more households are food insecure, fewer women deliver in a health facility, and fewer women are employed, as compared with Lagos. Mirroring these differences, this analysis identified that EIBF predictors unique to Kaduna included education level, household food security, health facility birth, and employment status. Cesarean birth is more common in Lagos than Kaduna, and, in Lagos, vaginal birth was one of the strongest predictors of EIBF.

Using composite scores not captured in past studies, we reported positive associations between EIBF practice and maternal knowledge and beliefs. In this analysis, formal education was positively associated with EIBF, in contrast to a study in several West African countries, which reported that mothers without formal education were more likely to practice EIBF [9]. The positive associations between EIBF and health facility birth [5,6,11,13,29], vaginal birth [6,7,9,13,14,46], and postpartum practices (such as avoiding prelacteal feeding) [6,17] have been reported previously in SSA. In Kaduna, up to 20% of infants are given prelacteal feeds, such as honey or chewed dates. Prelacteal feeding can delay the start of breastfeeding, as indicated in our analysis showing that EIBF is more common in infants who are not given prelacteal feeds. Our study also showed that the mother being head of household was positively associated with EIBF, which was likely related to their greater decision-making power as compared with mothers who were not household heads.

In both Lagos and Kaduna, maternal knowledge and self-efficacy were positively associated with EBF practice. In Kaduna, additional factors that were positively associated with EBF included education level, household food security, socioeconomic status, and the mother’s EBF awareness, beliefs, and postpartum practices. In Lagos, the mother’s perception of EBF norms was also positively associated with EBF. As with EIBF, these results likely reflect greater variability in education, household food security, and socioeconomic status in Kaduna as compared with Lagos.

To our knowledge, this is the first analysis to report associations between EBF and maternal awareness, beliefs, and norms using data at state level, because large national survey data do not typically collect information on beliefs and norms or have sufficient sample sizes for state-level EBF estimates. However, previous research has shown that maternal knowledge is positively associated with EBF [22], while inadequate EBF knowledge is negatively associated with the practice [25,26]. Our finding that household food security is positively associated with EBF aligns with qualitative and anecdotal findings from studies and programs about women’s perception that they cannot produce enough milk to exclusively breastfeed if they are not well nourished themselves [44,47,48]. Our results associating EBF with higher maternal education [19–21] and higher household socioeconomic or wealth status [19] align with findings from previous studies.

This analysis identified socioeconomic status as a common predictor of MDD practice across the two states. Other factors varied, likely reflecting substantial differences in food environments and sociocultural context. Kaduna has many rural, agricultural areas where own production may be an important component of diets and access to markets may be constrained, whereas Lagos is a very large urban area where nearly all food is purchased. Most of the associations with factors in the two states were in expected directions (e.g., receiving help from husband increases MDD). Exceptions were the negative association between MDD and secondary education in Kaduna and between MDD and food security in Lagos. In Kaduna, mothers who are more educated are more likely to be working away from home and leaving their children with alternate caregivers, who are often unable to provide a varied diet to young children [44]. In Lagos, food secure households are often those where the mother is employed and where more disposable income is available. These households tend to purchase prepared or packaged foods and snacks for young children [44], which may contribute to low dietary diversity.

Our findings that socioeconomic status [31,32,34,36], household food security [36], and mother’s nutrition knowledge [30] were positively associated with MDD were consistent with other studies. We found that contact with a health worker was associated with MDD. Similarly, other studies showed that ante- or postnatal health facility visits were related to MDD [34,35].

This analysis shows how predictors differ for each IYCF practice and are context dependent. Differences in predictors by IYCF practice are likely related, in part, to differences in the duration of each practice and in the attitudes and social norms that influence the behavior in each state. For example, mother’s status as the household head, which gives her decision-making power, and contact with a health worker, where mothers get trusted information, were predictors of EIBF in both states, while neither of these factors were predictive of EBF. These differences may be related to the one-time nature of EIBF as opposed to the sustained behavior and repeated decision-making required for EBF. Similarly, mothers receiving help related to child feeding from their husband or mother/mother-in-law predicted MDD in Kaduna, but not Lagos, and was not associated with EIBF or EBF in either state, although other studies have reported a link between social support and EBF [49–51]. In previous research we reported that women in Kaduna mainly need instrumental support (i.e., money) from their husbands to purchase complementary foods for their children and that women with support are more likely to practice MDD [52,53]. This aligns with the present finding of an association between support and MDD practice. Nearly all women in Lagos reported that their husbands help with MDD (and other IYCF practices). The lack of variability in this indicator may explain why there was no association between support and MDD in Lagos.

Across IYCF practices some combination of mother’s knowledge, awareness, beliefs, and self-efficacy were significantly associated with each outcome and dose-response relationships for these factors were generally observed, indicating that these factors are important parts of the potential pathways for behavior change. Previous analyses of data from Alive & Thrive evaluations in Nigeria and other countries have shown the importance of maternal knowledge, intentions, beliefs, norms, and self-efficacy in influencing IYCF practices. For example, Nguyen et al. [54] showed that exposure to the Alive & Thrive social and behavior change (SBC) intervention in Vietnam increased EBF by improving mothers’ knowledge, beliefs, social norms, and self-efficacy. In Nigeria, a study in Lagos showed that exposure to Alive & Thrive SBC in private health facilities increased women’s EBF knowledge and intentions [55].

This study had some limitations. We used cross-sectional data so we cannot infer causality in the relationships between factors assessed and specific IYCF practices. We found a larger number of predictors of IYCF practices in Kaduna than Lagos. This could be related to the larger sample size in Kaduna or could be explained by the greater variability in some of the factors in Kaduna. The items included in our knowledge and behavioral scores are not standardized but were adapted from baseline questionnaires used in Alive & Thrive impact evaluations in Bangladesh and Vietnam. It is possible other researchers would use different questions to create knowledge and behavioral scores, which could limit generalizability. IYCF practices are self-reported using the WHO IYCF questionnaire, so there is a possibility of social desirability bias. Our data were from the baseline of the Alive & Thrive evaluation, making it less likely that this bias was at play because the program had not yet been implemented. The analytic method we chose to create parsimonious scores resulted in different combinations of items in some cases in the two states (e.g., EIBF beliefs score), making the categories more adapted to each state, but less comparable across states. The strengths of this study were the large population-based samples in each state, which provided an opportunity to understand subnational differences in factors associated with IYCF practices. In addition, we collected data on behavioral and support factors that are not usually included in other analyses of predictors of IYCF.

In conclusion, context is critical to understanding factors associated with IYCF practices, as demonstrated by the subnational differences identified in this analysis. These details can inform the design and tailoring of interventions to improve IYCF practices in Nigeria. The inclusion of composite scores for maternal knowledge, awareness, beliefs, norms, self-efficacy, and postpartum practices sheds light on behavioral factors associated with optimal IYCF practices. Such factors have been understudied although they could potentially be modified though supportive interventions. We recommend that future research on factors associated with IYCF practices disaggregates subnationally, includes behavioral variables, and employs standardized definitions to facilitate comparisons. Understanding the factors associated with optimal IYCF practices, including those related to behaviors, beliefs, and social support, can inform future programs to improve child nutrition.

## Supporting information

S1 Table Survey questions used to create composite scores. (DOCX)

S2 Table Interactions in EIBF Model in Kaduna State. (DOCX)

S3 Table Interactions in EBF Model in Kaduna State. (DOCX)

S4 Table Interaction in MDD Model in Kaduna State. (DOCX)
