# Supplementary material for: Factors associated with infant and young child feeding practices in Kaduna and Lagos States, Nigeria
Source: PLOS Glob Public Health. 2025 Jun 27;5(6):e0004753. doi: 10.1371/journal.pgph.0004753 (PMC12204589; doi:10.1371/journal.pgph.0004753)
Supplement: S1 Table — (DOCX) [file pgph.0004753.s001.docx]

**S1 Table: Survey questions used to create composite scores.**

| **Composite score variable** | **Composite score questions** |
| --- | --- |
|  |  |
| **Knowledge composite score** | |
| EIBF (2 items) | What should a mother do with the “first milk” or colostrum? Give it to her baby soon after birth |
|  | How long after birth should a baby start breastfeeding? Immediately; Less than 1 hour after delivery |
| EBF (4 items) | When I say “exclusive breastfeeding,” what does that mean to you? Giving baby only breast milk and no other liquids or solids, not even water up to 6 months |
|  | What are some reasons a young baby should be exclusively breastfed? One or more correct responses.  Protects baby from illness  Helps baby grow better  Breast milk contains everything a baby needs for the first 6 months  Mother less likely to get pregnant  Delays return of mother’s monthly bleeding  Breast milk is clean and safe  Breast milk is convenient  Breast milk is affordable  Reduces health care costs |
|  | If a mother has a young baby (less than 6 months) and needs to be away from her baby and the baby gets hungry, what should the baby be fed? Mother’s expressed breast milk |
|  | What might happen if complementary food is started too early? One or more correct responses.  Causes the baby to breastfeed less  Results in a low nutrient diet  Increases the risk of illness  Increases a mother’s risk of pregnancy |
| MDD (9 items) | How many food groups should a child 6-23 months eat each day? Four or more |
|  | What foods does a young child (<24 months) need to grow and develop their brain? One point for each correct answer.  Pap with milk  Animal foods such as meat or chicken  Fish, shrimp, crab  Eggs  Fruits  Vegetables  Milk and dairy products  Peas/beans |
| **Awareness composite score** | |
| EIBF (2 items) | Have you ever heard about early initiation of breastfeeding within 1 hour after birth? |
|  | Have you ever heard about feeding children with colostrum? |
| EBF (1 item) | Have you ever heard about exclusive breastfeeding during the first 6 months (only breastmilk, no water)? |
| MDD (2 items) | Have you ever heard about starting complementary foods at 6 months of age? |
|  | Have you ever heard about feeding animal source foods (beef, chicken, fish, egg, organ meats)? |
| **Beliefs composite score^2^** | |
| EIBF (2 items) | If I breastfeed my infant within 1 hour after giving birth, it’ll be good for my child’s health |
|  | If I breastfeed my infant within 1 hour after giving birth, it’ll be good for my health |
| EBF (8 items) | If I am breastfeeding, but DO NOT give my infant water until s/he completes 6 months, my infant will be thirsty.^1^ |
|  | If I feed my infant ONLY breast milk and NO other food, water or infant formula until s/he completes 6 months, I am giving my infant all the nutrients s/he needs to be healthy. |
|  | If I am breastfeeding my 5-month-old infant, but DO NOT give my infant water, s/he will be too hot. |
|  | After I start to give infant formula to my infant, my body will produce less breast milk. |
|  | If I am breastfeeding and I wait until my infant has completed 6 months old to start feeding her/him semi-solid or solid foods, it is best for my infant s health. |
|  | If I feed my infant a combination of breast milk and other foods when s/he is between 4 and 6 months of age, I am giving my infant the best possible nutrition.^1^ |
|  | If I feed my infant ONLY breast milk and NO other food, water, or infant formula until he completes 6 months, I am giving my infant all the nutrients s/he needs for optimal brain development. |
|  | A mother who returns to work when her infant is 4 months old will have to use mainly formula to feed her infant. ^1^ |
| MDD (2 items) | If I give my infant organ meats like heart, liver, and kidney, starting at 7 months, it is good for his/her health. |
|  | If I feed my infant iron-rich food, such as meats, organ meats and dark green leafy vegetables starting at 6 months, it will help with brain development. |
| **Norms composite score^2^** | |
| EIBF (1 item) | Most mothers like me think that after normal delivery a mother can breastfeed her infant within 1 hour. |
| EBF (7 items) | Most mothers like me feed their babies only breast milk, and no other food, water, or infant formula for the first 6 months. |
|  | Most mothers like me approve of giving babies water before they reach 6 months of age. ^1^ |
|  | Most mothers like me give their babies water before 6 months of age. ^1^ |
|  | Most mothers like me approve of giving babies infant formula before they reach 6 months of age. ^1^ |
|  | Most mothers like me give their babies baby infant formula before 6 months of age. ^1^ |
|  | Most mothers like me approve of giving babies semi-solid or solid foods before they reach 6 months of age. ^1^ |
|  | Most mothers like me give their babies semi-solid or solid foods before 6 months of age.^1^ |
| MDD (2 items) | Most mothers like me add egg or fish or liver or meat or chicken in addition to other foods every day, starting at 6 months. |
|  | Most mothers like me feed their babies fruits and vegetables every day beginning at 6 months of age. |
| **Self-efficacy composite score^2^** | |
| EIBF (2 items) | How confident were you about making decisions about what you have fed your baby in the first few hours and days after delivery? |
|  | How confident were you about not feeding your baby prelacteals but only colostrum in the first few days after delivery? Prelacteal feeds are those liquids or foods given to newborns before breastfeeding for the first time. |
| EBF (3 items) | How confident are/were you that you will be/were able to give only breastmilk to your baby in the first 6 months without even giving a drop of water? |
|  | How confident are/were you that you will be/were able to sufficiently produce breast milk to meet the child’s nutritional need in the first 6 months? |
|  | How confident are/were you that you will be/were able to prevent anyone trying to feed your baby anything other than breastmilk, like purchased baby foods (e.g. infant formula, pap, or other food/drinks), before 6 months of age? |
| MDD (2 items) | How confident are/were you that you will be/were able to feed your baby mashed family cooked foods (rice, yam, vegetable) along with breastmilk after 6 months of age? |
|  | How confident are/were you that you will be/were able to feed your baby animal source foods (such as egg, fish, and chicken liver) along with breastmilk after 6 months of age? |
| **Postpartum practices composite score** | |
| EIBF (2 items) | Did anyone put anything inside your baby’s mouth IMMEDIATELY after the birth? ^1^ |
|  | Did you or anyone else check to see if your baby was sucking well? |
| EBF (3 items) | Did anyone put anything inside your baby’s mouth IMMEDIATELY after the birth? ^1^ |
|  | Did you or anyone else check to see if your baby was sucking well? |
|  | During the first 3 days after your baby was born, what was put into the child’s mouth by you or anyone else? Nothing was put in the child’s mouth |
| **Mother’s complementary food decision making composite score** | |
| MDD (2 items) | Who usually makes decisions about what foods to purchase for your child: you, your husband/partner, or you and your husband/partner jointly? |
|  | Who usually makes decisions about what to feed your child: you, your husband/partner, or you and your husband/partner jointly? |

^1^ Responses were recoded to go in a positive direction for the composite summation score.

^2^Responses to beliefs, norms, and self-efficacy questions were recorded using a 5-point scale. The responses were recoded so that somewhat agree/somewhat confident and strongly agree/strongly confident were coded as one and the other three response options were coded as zero.
