# Supplementary material for: Factors associated with infant and young child feeding practices in Kaduna and Lagos States, Nigeria
Source: PLOS Glob Public Health. 2025 Jun 27;5(6):e0004753. doi: 10.1371/journal.pgph.0004753 (PMC12204589; doi:10.1371/journal.pgph.0004753)
Supplement: S2 Table — (DOCX) [file pgph.0004753.s002.docx]

**S2 Table: Interactions in EIBF Model in Kaduna State.**

|  | **0-3 ANC visits** | **4+ ANC visits** |
| --- | --- | --- |
|  | **OR** | **OR** |
| Mother’s EIBF knowledge |  |  |
| 0 items | 1.00 | 0.71 |
| 1 item | 0.95 | 0.05 |
| 2 items | 12.69 | 8.98 |

|  | **Postpartum**  **practices**  **0 items** | **Postpartum**  **practices**  **1 item** | **Postpartum**  **practices**  **2 items** |
| --- | --- | --- | --- |
|  | **OR** | **OR** | **OR** |
| Mother’s EIBF knowledge |  |  |  |
| 0 items | 1.00 | 0.97 | 0.99 |
| 1 item | 0.95 | 2.56 | 0.94 |
| 2 items | 12.69 | 12.27 | 12.55 |

|  | **EIBF self-efficacy**  **0 items** | **EIBF self-efficacy**  **1 item** | **EIBF self-efficacy**  **2 items** |
| --- | --- | --- | --- |
|  | **OR** | **OR** | **OR** |
| Mother’s EIBF knowledge |  |  |  |
| 0 items | 1.00 | 0.86 | 2.70 |
| 1 item | 0.95 | 0.82 | 2.58 |
| 2 items | 12.69 | 10.95 | 27.14 |

|  | | **Food Secure** | | **Food Insecure** |
| --- | --- | --- | --- | --- |
|  | | **OR** | | **OR** |
| Mother’s EIBF self-efficacy | |  | |  |
| 0 items | | 1.55 | | 1.00 |
| 1 item | | 0.98 | | 0.86 |
| 2 items | | 4.19 | | 2.70 |
|  |  | |  | |
