# Supplementary material for: Factors associated with infant and young child feeding practices in Kaduna and Lagos States, Nigeria
Source: PLOS Glob Public Health. 2025 Jun 27;5(6):e0004753. doi: 10.1371/journal.pgph.0004753 (PMC12204589; doi:10.1371/journal.pgph.0004753)
Supplement: S3 Table — (DOCX) [file pgph.0004753.s003.docx]

**S3 Table: Interactions in EBF Model in Kaduna State.**

|  | **Mother’s EBF knowledge**  **0-2 items** | **Mother’s EBF knowledge**  **3+ items** |
| --- | --- | --- |
|  | **OR** | **OR** |
| No help from mother or mother-in-law | 1.00 | 4.40 |
| Any help from mother or mother-in-law | 1.15 | 1.44 |

|  | **Mother’s EBF beliefs**  **0-2 items** | **Mother’s EBF beliefs**  **3-4 items** | **Mother’s EBF beliefs**  **5-8 items** |
| --- | --- | --- | --- |
|  | **OR** | **OR** | **OR** |
| Mother’s EBF self-efficacy |  |  |  |
| 0 items | 1.00 | 1.56 | 1.72 |
| 1 item | 1.84 | 2.87 | 3.16 |
| 2 items | 3.19 | 4.98 | 18.53 |
