# Supplementary material for: Factors associated with infant and young child feeding practices in Kaduna and Lagos States, Nigeria
Source: PLOS Glob Public Health. 2025 Jun 27;5(6):e0004753. doi: 10.1371/journal.pgph.0004753 (PMC12204589; doi:10.1371/journal.pgph.0004753)
Supplement: S4 Table — (DOCX) [file pgph.0004753.s004.docx]

**S4 Table: Interaction in MDD Model in Kaduna State.**

|  | **Not breastfeeding** | **Still breastfeeding** |
| --- | --- | --- |
|  | **OR** | **OR** |
| Mother’s education level |  |  |
| None to primary | 1.00 | 1.72 |
| Some secondary or higher | 0.74 | 2.86 |
